# Characterization and Protective Efficacy of a *Salmonella Typhimurium* ATCC 14028 *sptP* Mutant as a Live Attenuated Vaccine Candidate

**DOI:** 10.3390/vaccines13020150

**Published:** 2025-01-31

**Authors:** Nanlong Zhou, Yonghui Ding, Ting He, Yuling Sun, Hongfang Chen, Meiling Huang, Tiansen Li

**Affiliations:** 1 School of Tropical Agriculture and Forestry, Hainan University, Haikou 570100, China; nanlongzhou@163.com (N.Z.);; 2 Key Laboratory of Tropical Translational Medicine of Ministry of Education, Hainan Key Laboratory for Research and Development of Tropical Herbs, School of Pharmacy, Hainan Medical University, Haikou 571199, China

**Keywords:** *Salmonella Typhimurium*, *sptP*, live attenuated vaccine, mutant, immunogenicity

## Abstract

Background: *Salmonella Typhimurium* poses a substantial health risk to both humans and animals. This study evaluated the potential of using the *Salmonella Typhimurium* Δ*sptP* mutant as a live-attenuated vaccine candidate by constructing it through homologous recombination and assessing its key biological properties, including growth characteristics, immunogenicity, and protective efficacy. Methods: We generated the Δ*sptP* mutant through targeted gene deletion, ensuring the preservation of the bacterial strain’s growth and stability. In vitro and in vivo assays were performed to compare the invasive capabilities between the mutant and the wild-type strains. Specifically, we examined the invasion into RAW264.7 murine macrophages and mice. Furthermore, the virulence of the mutant was evaluated by determining the median lethal dose (LD_50_). To evaluate immunogenicity and protection, mice were immunized with 2 × 10^4^ CFUs of the Δ*sptP* mutant, followed by a booster immunization, and then challenged with a virulent strain. Results: The Δ*sptP* mutant exhibited no significant changes in growth characteristics or genetic stability compared to the wild-type strain. However, it demonstrated a significantly diminished capacity for invasion in both murine macrophages and mice. The LD_50_ for the mutant was 39.92-fold higher than that of the wild-type, indicating a marked reduction in virulence. Mice immunized with the Δ*sptP* mutant and administered a booster immunization exhibited 87.5% protection against challenge with a virulent strain, as compared to the PBS control group. Moreover, the mutant induced IgG antibody levels comparable to those induced by the wild-type strain. Conclusions: The Δ*sptP* mutant of *Salmonella Typhimurium* exhibits markedly reduced virulence while retaining robust immunogenicity and protective efficacy. These findings suggest that the Δ*sptP* mutant is a promising candidate for a live-attenuated vaccine, potentially providing an effective strategy to prevent *Salmonella Typhimurium* infections.

## 1. Introduction

*Salmonella Typhimurium*, a Gram-negative bacterium, is widely distributed across various environments, including livestock and wild-animal populations [1]. This bacterium exhibits conditional pathogenicity and poses a substantial health risk to humans and domestic animals by being transmitted through contaminated food and water sources [2]. Infection with *Salmonella Typhimurium* can cause a range of intestinal diseases in both animals and humans [3], causing systemic symptoms such as gastroenteritis and septicemia, as well as tissue and organ damage [4]. Such infections not only increase livestock mortality but also reduce productivity and growth rates, resulting in significant economic losses for the agricultural sector [5].

*Salmonella Typhimurium* primarily spreads through the fecal–oral route, invades the host [6], and subsequently colonizes the host’s macrophages [5]. By utilizing a distinct array of unique virulence factors and typhoid toxins, the infection symptoms in the host progressively develop, and virulence proteins interact with the host cells to facilitate bacterial survival and proliferation within them [7,8,9]. The region of the *Salmonella* genome encoding virulence-related genes, known as the Salmonella Pathogenicity Island (SPI) [10], which plays a crucial role in *Salmonella*’s invasion and disease processes. Notably, SPI-1 and SPI-2 are closely linked to its pathogenic potential [11,12].

Currently, antibiotics remain a highly effective means for controlling *Salmonella Typhimurium* infections. However, their prolonged use has raised growing concerns regarding antibiotic residues and the development of bacterial resistance [13,14,15]. As a result, vaccination has emerged as an effective preventive strategy against *Salmonella Typhimurium* infection [16]. With the expanding body of research into *Salmonella Typhimurium*’s pathogenic mechanisms, the engineering of attenuated strains by deleting virulence genes is becoming increasingly prevalent in the development of such vaccines [17,18,19,20]. The SptP protein, a critical virulence factor of *Salmonella Typhimurium* [21,22], triggers the reorganization of the host cell actin cytoskeleton upon bacterial entry, thereby facilitating invasion [23,24]. SptP exerts a crucial regulatory role in the intracellular survival of *Salmonella Typhimurium*.

Consequently, we constructed a *sptP* gene mutant of *Salmonella Typhimurium* using the homologous recombination technique. Subsequently, we characterized the mutant strain’s biological properties, such as growth dynamics, immunogenic potential, and invasive capacity, and preliminarily assessed its protective efficacy to ascertain whether the *sptP* gene deletion impacts the bacterium’s virulence. It is essential to research and develop live attenuated vaccines to prevent and control *Salmonella Typhimurium* infection.

## 2. Materials and Methods

### 2.1. Bacterial Strains, Plasmids, and Primers

The wild-type strain of *Salmonella Typhimurium* ATCC14028 used in this study was maintained in our laboratory. The *sptP* gene mutant (ATCC14028Δ*sptP*) was constructed via homologous recombination using plasmids pACYC184 and pKD46, together with the primers *sptP*-cat-F and *sptP*-cat-R. Strains were cultured in Luria–Bertani (LB) broth containing 1% (wt/vol) tryptone, 0.5% (wt/vol) yeast extract, and 0.5% (wt/vol) NaCl, or on LB agar plates with an additional 1.5% (wt/vol) agar, depending on the experimental needs. When required, ampicillin (Amp, 50 µg/mL) or chloramphenicol (Cm, 25 µg/mL) was supplemented into the medium. The sequences of the primers used in this study are provided in Table 1.

### 2.2. Construction of the sptP Mutant

The *sptP* gene mutant (ATCC14028Δ*sptP*) was constructed using the suicide vector pKD46 through homologous recombination. Briefly, the chloramphenicol resistance cassette was PCR-amplified from pACYC184 using the primers *sptP*-cat-F and *sptP*-cat-R, which incorporated 50 bp homology arms at both the 5′ and 3′ ends of the *sptP* locus. The purified PCR product was then introduced into competent *Salmonella Typhimurium* cells containing pKD46 by electroporation. Single-crossover mutants were selected on chloramphenicol-containing LB agar plates and verified by PCR using the primers *sptP*-out-F and *sptP*-out-R. Sequencing further confirmed the successful disruption of the *sptP* gene.

### 2.3. Identification of the Biological Characteristics of ATCC14028ΔsptP In Vitro

To evaluate the growth characteristics of the ATCC14028Δ*sptP* mutant, we performed colony morphology analysis and biochemical identification on MacConkey agar plates. The findings were then compared with those of the wild-type ATCC14028 strain. Following this, the growth kinetics and genetic stability of both strains were examined. Both the wild-type ATCC14028 and ATCC14028Δ*sptP* strains were cultured under conditions of 180 rpm agitation at 37 °C, with optical density (OD_600_) measurements recorded every 2 h over a 36 h period. Under these conditions, the ATCC14028Δ*sptP* strain was passaged for up to 30 generations. Samples from the 5th, 10th, 15th, 20th, 25th, and 30th passages were collected for PCR verification using the primers *sptP*-out-F and *sptP*-out-R, followed by sequencing to confirm genetic stability.

### 2.4. The Ability of Bacteria to Invade Cells

Mouse macrophage RAW264.7 cells were grown in DMEM (high glucose) supplemented with 10% fetal bovine serum (FBS) and antibiotics (50 µg/mL penicillin). The cells were incubated at 37 °C in a humidified atmosphere of 5% CO_2_ and 95% air. When the cells reached 90% confluence, ATCC14028 and ATCC14028Δ*sptP* strains, which were normalized by OD_600_, at a multiplicity of infection (MOI) of 100:1 for 1 h.

After infection, extracellular bacteria were eliminated by washing the cells three times with PBS, followed by a 1 h incubation in DMEM containing 100 µg/mL gentamicin to eliminate residual extracellular bacteria. At designated post-infection time points (0, 2, 4, 8, 12, 24, and 48 h), the infected cells were washed three times with PBS and lysed with 1% Triton X-100 (wt/vol). The lysates were serially diluted, plated on MacConkey agar, and incubated at 37 °C for 12 h, after which colony-forming units (CFUs) were enumerated.

### 2.5. Assessment of Bacterial Virulence

Female BALB/c mice, aged 4–5 weeks, were housed under specific pathogen-free (SPF) conditions in our laboratory. All animal experiments were conducted in accordance with protocols approved by the Animal Protection and Ethics Committee of Hainan University (Haikou, China). To evaluate the virulence of ATCC14028Δ*sptP* in mice, seventy mice were randomly assigned into fourteen groups, each comprising five mice. Each group was administered an intraperitoneal inoculation of ATCC14028Δ*sptP* at one of ten serial dilutions, ranging from 1 × 10^5^ to 1 × 10^10^ CFUs, each in 200 μL of PBS. In a control experiment, ten additional mice were inoculated intraperitoneally with 200 μL of PBS. Mortality rates were recorded for each group at day 14 post-challenge. The median lethal dose (LD_50_) for the two strains was determined using the modified Cole method.

### 2.6. Histopathological Tests

Surviving mice were humanely euthanized, and their livers and spleens were excised and immediately fixed in 4% paraformaldehyde. After fixation, tissue samples were processed for paraffin embedding, sectioned, stained with hematoxylin and eosin (H&E), and subjected to histopathological evaluation under an optical microscope.

### 2.7. Bacterial Invasion Ability Test

Seventy female BALB/c mice (4–5 weeks old) were randomly allocated into three groups: the wild-type strain group, the Δ*sptP* mutant group, and the PBS control group. Each mouse was injected intraperitoneally with 2 × 10^4^ CFUs of the respective inoculum. On days 3, 6, 9, and 12 post-infection, mice were humanely euthanized, and liver, spleen, lung, and kidney samples were aseptically harvested. Tissue samples were homogenized using a high-throughput tissue homogenizer (KZ-III-FP, Wuhan Servicebio Biotechnology Co., Ltd., Wuhan, China) in sterile PBS. Homogenate dilutions were plated on McConkey agar and incubated at 37 °C for 12–16 h to quantify CFUs.

### 2.8. Immunoprotective Assessment of ATCC14028ΔsptP

The immunoprotective effect of ATCC14028Δ*sptP* in mice was assessed through intraperitoneal injections. Eighteen female BALB/c mice (4–5 weeks old) were randomly divided into three groups (*n* = 6 per group): the immunized group, the non-immunized group, and the PBS control group. Mice in the immune group received an intraperitoneal injection of 2 × 10^4^ CFUs of ATCC14028Δ*sptP*, while the nonimmune and blank control groups were administered 200 μL of sterile PBS each. Two weeks post-initial immunization, booster doses were administered twice at one-week intervals. Following two weeks after the booster immunization, both the immunized and nonimmunized groups received an intraperitoneal injection of wild-type ATCC14028 at 10 times the LD_50_ (5.01 × 10^6^ CFUs), and the blank control group received 200 μL of PBS in a similar manner. Morbidity and survival rates were monitored for two weeks post-challenge, and liver and spleen tissues from surviving mice were collected for histopathological analysis.

### 2.9. Serum IgG Assay

Mice were immunized via intraperitoneal injection following the procedure and dosage outlined in the immunoprotective efficacy evaluation. Blood samples were collected from the mice via retro-orbital puncture using capillary tubes, and serum was isolated through centrifugation. Changes in IgG levels were quantified using a mouse *Salmonella* IgG ELISA kit (Jiangsu Meimian Industrial Co., Ltd., Jiangsu, China) with absorbance measured at 492 nm.

### 2.10. Data Analysis

Data analysis was conducted using GraphPad Prism 8 software (version 8.4.3). Data are presented as means ± SEM. Within each group, an independent sample T-test was applied, while an analysis of variance for repeated measures (ANOVA) was used to compare means between groups. (* *p* < 0.05; ** *p* < 0.01; *** *p* < 0.001).

## 3. Results

### 3.1. Construction and Biological Characteristics of Mutant ATCC14028ΔsptP

**Figure 1:**
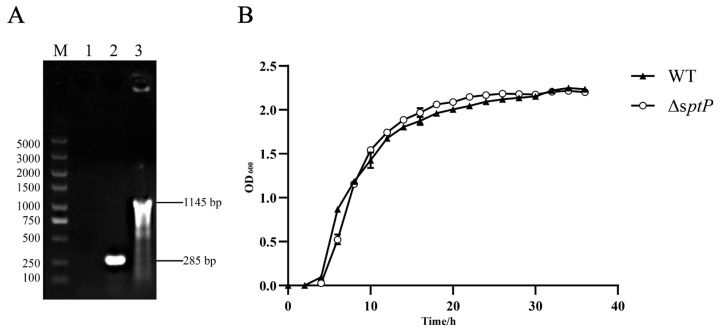
Construction and biological characteristics of mutant ATCC14028Δ*sptP*. (**A**) PCR validation of lateral primers. M, DL5000 DNA Marker; Lane 1: negative control; Lane 2: wild-type strain ATCC14028 genomic DNA; Lane 3: ATCC14028Δ*sptP* genomic DNA. The PCR product for wild-type ATCC14028 measured 285 bp, while the ATCC14028Δ*sptP* mutant, incorporating a chloramphenicol resistance gene, extended to 1145 bp. (**B**) Growth curves of ATCC14028 and its mutant ATCC14028Δ*sptP*, cultured in LB medium at 37 °C and 180 rpm for 36 h, with optical density at 600 nm (OD_600_) measured bi-hourly.

A mutant strain of *Salmonella Typhimurium* ATCC14028 with a disrupted *sptP* gene was generated through homologous recombination. PCR identification of ATCC14028Δ*sptP*, performed using the *sptP*-cat-F/*sptP*-cat-R primers pair, confirmed the successful insertion of the chloramphenicol resistance gene into the *sptP* gene fragment. The mutant and wild-type strains were differentiated via PCR using flanking primers *sptP*-out-F and *sptP*-out-R. The presence of 1145 bp and 285 bp amplicons confirmed the successful construction of ATCC14028Δ*sptP* (Figure 1A). Additionally, the genetic stability and identity of the mutant were confirmed by DNA sequencing (Supplementary File, Figure S1A). Growth kinetics data indicated no statistically significant disparities in the growth rates of the mutant strain compared to the wild-type strain when cultivated at 37 °C in LB medium (Figure 1B). Similarly, biochemical characterization showed no significant differences between the mutant and wild-type strains (Supplementary File, Table S1).

### 3.2. Mutations in sptP Reduce Bacterial Invasion in Cells

**Figure 2:**
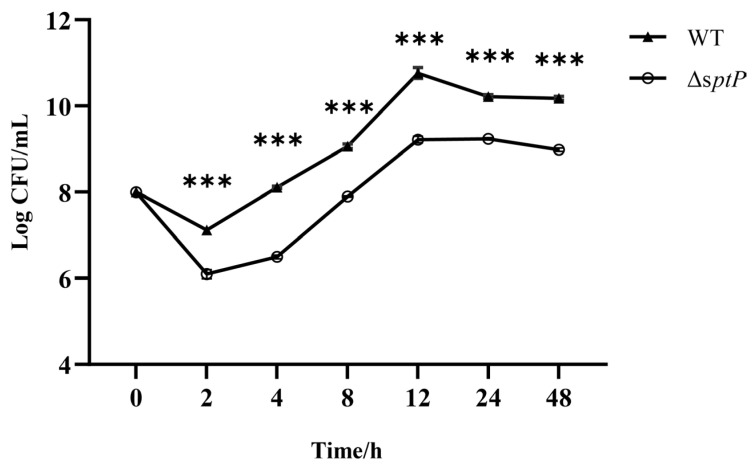
Invasion of ATCC14028 and ATCC14028Δ*sptP* in RAW264.7 mouse macrophages. Bacterial counts were measured, and the results were expressed as Log CFU/mL. Data are presented as the mean ± SEM. *** *p* <0.001.

Bacterial invasion in of RAW264.7 murine macrophages were quantified using a gentamicin protection assay. Quantitative analysis revealed that bacterial counts of ATCC14028Δ*sptP* in cells were significantly lower than those of the wild-type strain at all time points during the infection (Figure 2, *p* ≤ 0.001). These findings suggest that disruption of the *sptP* gene impairs the intracellular invasion capability of *Salmonella Typhimurium*.

### 3.3. ATCC14028ΔsptP Showed Reduced Virulence in Mice

To assess the virulence of ATCC14028Δ*sptP* in mice, both ATCC14028Δ*sptP* and the wild-type strain were administered intraperitoneally, and mouse survival was monitored. Analysis showed that the LD_50_ of ATCC14028Δ*sptP* was 2 × 10^7^ CFUs, which is 40 times higher than that of the wild-type strain (5.01 × 10^5^ CFUs). This indicates that disruption of the *sptP* gene significantly reduces the virulence of *Salmonella Typhimurium* (Table 2).

### 3.4. ATCC14028ΔsptP Showed Fewer Pathological Changes than Wild-Type Strains

**Figure 3:**
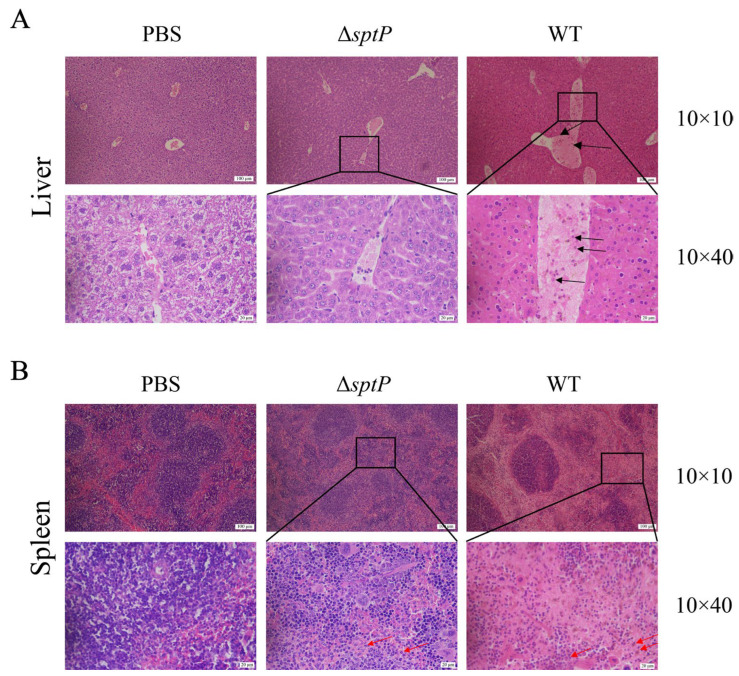
Histological analysis of mice after intraperitoneal injection of ATCC14028 and ATCC14028Δ*sptP*. At 14 days, histopathological changes in the liver (**A**) and spleen (**B**) were detected by H&E staining. The results were observed using an optical microscope at magnifications of 100× and 400×. Note: The black and red arrows indicate inflammatory cells.

H&E staining and histological analysis of liver and spleen tissues from challenged mice revealed that, compared to the PBS control group, the wild-type group exhibited notably dilated hepatic veins with signs of congestion and hemorrhage, as well as a significant infiltration of inflammatory cells into the vessels and surrounding tissue. In the Δ*sptP* group, there were minimal inflammatory cells, and no other lesions observed (Figure 3A). Relative to the PBS control group, spleen nodules in the wild-type group were significantly enlarged, accompanied by extensive inflammatory cell exudation and bleeding. The Δ*sptP* group exhibited milder symptoms, characterized by slight enlargement of splenic nodules, minimal inflammatory cell exudation, and no additional lesions (Figure 3B). Collectively, these findings suggest that the *sptP* mutation in *Salmonella Typhimurium* results in a more attenuated disease phenotype in infected mice.

### 3.5. The Invasion Capacity of ATCC14028ΔsptP in Mouse Visceral Tissues Was Reduced Compared to the Wild-Type Strain

The CFUs counts in the liver, spleen, lungs, and kidneys of mice were determined following injection with ATCC14028Δ*sptP* and the wild-type ATCC14028 strain. In the third day post-injection, ATCC14028Δ*sptP* exhibited a decreasing trend in organ invasion, in contrast to the increasing trend observed in wild-type strains. The replication of ATCC14028Δ*sptP* peaked on day 6 post-injection and then declined. Notably, the clearance rate of ATCC14028Δ*sptP* within the first three days was significantly higher compared to wild-type strains, and its peak invasion level on the sixth day was lower than that observed in wild-type strains.

**Figure 4:**
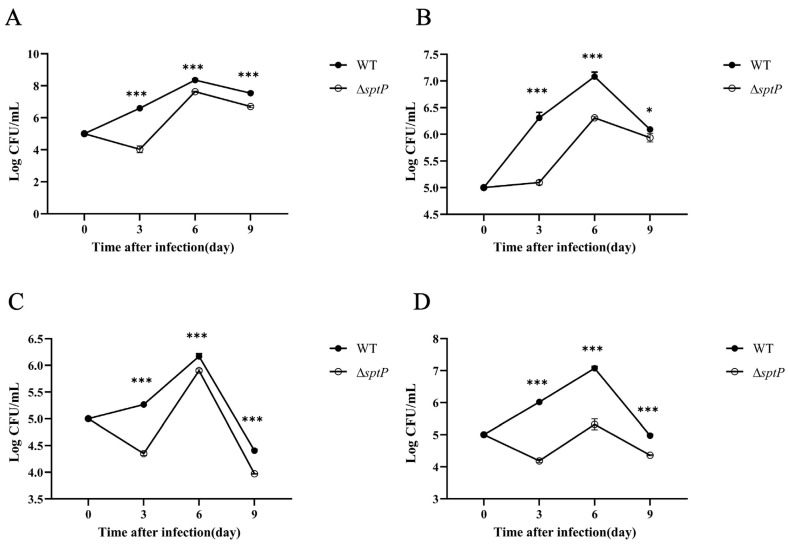
The invasion capacity of ATCC14028 and ATCC14028Δ*sptP* in mouse organs. Bacterial invasion and persistence in the liver (**A**), spleen (**B**), lungs (**C**), and kidneys (**D**) of mice after intraperitoneal injection of ATCC14028 and ATCC14028Δ*sptP* at 2 × 10^4^ CFUs. Bacterial counts were measured, and the results were expressed as Log CFU/mL. Data are presented as the mean ± SEM, where * denotes significance levels: * *p* < 0.05, and *** *p* < 0.001.

Interestingly, on the sixth day post-injection, the CFU counts for both the mutant and wild-type strains were more similar in liver and lung tissues compared to spleen and kidney tissues. Specifically, while the wild-type strain maintained a higher bacterial load in all organs, the difference in CFU counts between the mutant and wild-type strains was less pronounced in the liver and lungs relative to the spleen and kidneys. This suggests that the *sptP* gene mutation has a differential impact on bacterial survival and replication across different host tissues. These findings demonstrate that the *sptP* gene mutation significantly diminishes the infection and invasion capabilities of *Salmonella Typhimurium* in mice (Figure 4). The reduced ability of the ATCC14028Δ*sptP* to persist in major organs highlights the critical role of the *sptP* gene in facilitating systemic infection and tissue-specific colonization.

### 3.6. ATCC14028ΔsptP Protects Mice Against Wild-Type Salmonella Typhimurium

**Figure 5:**
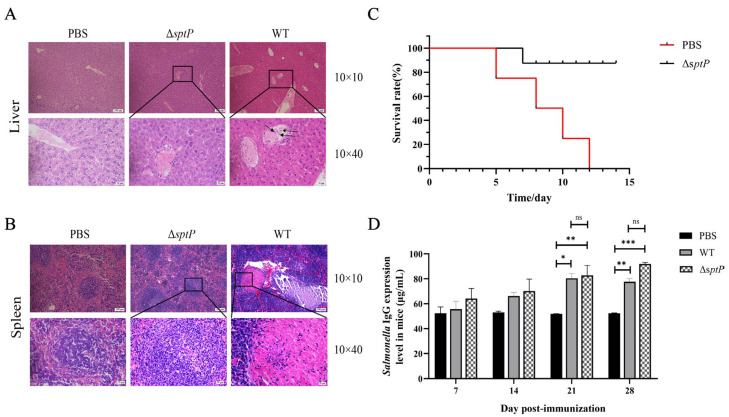
Immunoprotective effect and antibody levels of ATCC14028Δ*sptP*. Histopathological changes in the liver (**A**) and spleen (**B**) after immunization with ATCC14028Δ*sptP* were detected via H&E staining. (**C**) Survival rates of mice immunized with ATCC14028Δ*sptP* post-challenge. (**D**) Testing of serum IgG expression levels in mice. Data are presented as the mean ± SEM. * *p* ≤ 0.05, ** *p* ≤ 0.01, *** *p* <0.001, and ns indicates no significant difference (*p* ≥ 0.05).

Following two immunizations with ATCC14028Δ*sptP*, the survival rates of mice after challenge with virulent strains are presented in Figure 5C. In the PBS control group, mortalities were observed on days 5, 8, 10, and 12 post-infection (DPI). Two additional deaths occurred on day 9 DPI in the PBS control group following challenge with the wild-type strain. One death was recorded on day 7 DPI in the ATCC14028Δ*sptP*-immunized group. These results indicate that immunization with the Δ*sptP* strain of *Salmonella Typhimurium* significantly protects against subsequent challenge with virulent strains, achieving an 87.5% protection rate. At the end of the experiment, only one mouse survived in the wild-type strain group inoculated with 1 × 10^6^ CFUs, making the sample size too small for meaningful statistical analysis and pathological observation. Therefore, to ensure a sufficient sample size and reliable results, we chose to perform histopathological examinations on surviving mice from the 1 × 10^5^ CFUs concentration groups for both the wild-type and mutant strains. Histopathological evaluation revealed that, compared to the PBS control group, the wild-type group exhibited dilated hepatic veins, markedly enlarged splenic nodules, and a significant presence of inflammatory cells within blood vessels. In contrast, the ATCC14028Δ*sptP* group showed no significant histopathological alterations (Figure 5A,B).

### 3.7. The ATCC14028ΔsptP Strain Induced an Immune Response Similar to That of the Wild-Type Strain

IgG levels in mice were measured following immunization (Figure 5D). In the first and second weeks post-initial immunization, there were no significant differences in IgG levels between the ATCC14028Δ*sptP* and wild-type groups compared to the PBS control group. However, after booster immunizations, IgG levels significantly increased in both the Δ*sptP* strain and wild-type groups, with no substantial difference between them. These findings suggested that the Δ*sptP* strain of *Salmonella Typhimurium* can elicit an immune response comparable to that induced by the wild-type strain.

## 4. Discussion

*Salmonella Typhimurium*, a prevalent intestinal pathogen, is recognized for causing a range of gastrointestinal illnesses in both animals and humans, which can result in persistent infections and subsequently lead to systemic symptoms and tissue damage [25,26,27]. Given the rising antibiotic resistance in *Salmonella* spp. and the lack of new antibiotics [28], attenuated *Salmonella* strains provide an ideal vaccine strategy for infection control and prevention. Multiple live attenuated vaccines against Salmonella have been developed, showing greater efficacy than inactivated vaccines [29]. Attenuated *Salmonella Typhimurium* strains can reduce or eliminate virulence by various means of knocking out virulence genes, but these strains possess inherent shortcomings, such as the potential reversion to pathogenicity due to gene repair, thus limiting the clinical use of such live attenuated vaccines [30].

In this study, we successfully engineered a mutant strain of *Salmonella Typhimurium* ATCC14028Δ*sptP* using homologous recombination. Vaccine strains require a well-defined genetic background and genetic stability, which are critical for developing effective attenuated vaccines. The Δ*sptP* mutant strain maintained the *sptP* gene mutation stably over 30 consecutive generations. Moreover, we verified that mutating the *sptP* gene did not significantly affect colony morphology, biochemical characteristics, or growth rate, likely due to its acquisition through horizontal gene transfer linked to SPI-1 [31]. These findings support the potential of the *sptP* gene as a target for live attenuated vaccine development.

We subsequently evaluated the Δ*sptP* mutants for safety, serum immunoglobulin levels, and protective efficacy, revealing the *sptP* gene’s critical role in *Salmonella Typhimurium* pathogenicity and providing robust evidence for the potential of ATCC14028Δ*sptP* as an effective live attenuated vaccine against *Salmonella Typhimurium* infections. Although oral vaccination can mimic the practical application of vaccines in livestock and humans, we opted for intraperitoneal injection primarily due to the following considerations: Firstly, intraperitoneal injection allows for precise control over the vaccine dose’s accuracy and consistency, crucial for initial safety and immunogenicity assessments [32,33]. Secondly, this approach avoids confounding factors from the digestive tract during the initial stages, enabling the collection of direct and unobstructed immune response data. Literature also indicates that in the initial evaluation stage of vaccines, non-oral routes are a common practice, especially for exploring the basic immunogenicity and efficacy of vaccines [33,34,35]. Although oral administration is closer to the actual application scenario of the vaccine, the current research aims to confirm the core safety and immune effect of the vaccine by intraperitoneal injection, avoiding the complex variables that may be introduced by digestive enzymes, mucosal barrier variability, and other factors in the early stage. Once the vaccine’s basic immunogenicity and safety are established, our research will transition smoothly to the oral route to simulate vaccine application accurately in the target species. This step-by-step strategy not only ensures systematic and phased research, but also follows the traditional path of vaccine research and development [36,37].

Our findings revealed that mutating the *sptP* gene in *Salmonella Typhimurium* significantly reduced bacterial viability in the mutant strain relative to the wild-type strain during infection of RAW264.7 macrophages. This reduction was particularly evident in the early stages of infection, indicating that *sptP* plays a crucial role in facilitating bacterial entry into host cells during the initial infection phase. Moreover, the ability of the Δ*sptP* mutant strain to colonize organs in mice was markedly diminished. These findings suggest that *sptP* effector molecules play a key role in enhancing the invasion capabilities of *Salmonella Typhimurium* into host cells. Essential characteristics of live attenuated vaccines include safety and non-toxicity.

In our study, after intraperitoneal administration of the Δ*sptP* mutant and wild-type strains to separate groups of BALB/c mice, we found that the calculated LD_50_ value of the Δ*sptP* mutant strain was found to be 39.92-fold higher than that of the wild-type strain. This indicates a significant reduction in the virulence of the Δ*sptP* mutant. In addition to attenuation, it is crucial for live attenuated vaccines to exhibit no adverse effects on host animals. Immunization with the Δ*sptP* mutant resulted in only minor histopathological changes in the inoculated mice. Concurrently, the wild-type strain induced significant infiltration of inflammatory cells into the liver and spleen, along with symptoms of congestion, demonstrating that the mutant is sufficiently safe and attenuated for use as a live vaccine.

In the context of live attenuated vaccines, immune protection is a pivotal criterion for evaluating vaccine efficacy. Consequently, live attenuated vaccines effectively elicit host-specific humoral and cellular immune responses, which are critical for preventing secondary infections by pathogens [38]. After immunization with the Δ*sptP* mutant strain, serum IgG levels measured on days 21 and 28 were comparable to those induced by the wild-type strain and significantly higher than those in the PBS control group. Furthermore, the Δ*sptP* mutant strain provides up to 87.5% immune protection against a virulent strain of *Salmonella Typhimurium*. Mice immunized with the Δ*sptP* mutant strain exhibited no significant pathological changes in the liver and spleen post-infection with the wild-type strain, unlike those in the PBS control group. Therefore, while the virulence of *Salmonella Typhimurium* is attenuated following the *sptP* gene mutation, it maintains commendable immunogenicity, enabling it to confer a robust level of both humoral and cellular immunity and protect the host.

Given the potential real-world applications of this vaccine, it is crucial to approach its associated risks cautiously. Firstly, although our studies have demonstrated that the Δ*sptP* mutant exhibits reduced virulence and genetic stability under laboratory conditions, these characteristics may differ in complex natural environments. Long-term monitoring of the vaccine strain is essential to ensure its safety, especially when evaluating its performance across diverse host populations. Different types of animals may exhibit varied immune responses to the same vaccine [17]. Additionally, considering the potential for live vaccine strains to spread in the environment, it is imperative to carefully assess the impact of such dissemination on ecosystems and wildlife populations [39]. Implementing appropriate measures, such as optimizing administration methods and dosages, to limit exposure to non-target species is crucial for maintaining ecological balance.

Overall, this study provides a robust theoretical foundation for the development of an attenuated live vaccine for *Salmonella Typhimurium*. Future work will continue to focus on enhancing the safety and efficacy of the vaccine, aiming to provide a safe and effective solution for preventing *Salmonella Typhimurium* infections.

## 5. Conclusions

In summary, we successfully engineered a mutant strain of *Salmonella Typhimurium* with a Δ*sptP* gene deletion. Experimental results showed that the invasive capability and overall virulence of *Salmonella Typhimurium* were significantly reduced following the *sptP* gene mutation. Moreover, the Δ*sptP* mutant elicited strong immunogenic responses and provided robust immune protection in mice, indicating its potential as an effective live attenuated vaccine against *Salmonella Typhimurium*. These findings lay a solid theoretical foundation for further exploring and developing live attenuated vaccines.

## Figures and Tables

**Table 1:** Primers used in this study.

Primers	Sequences (5′-3′) ^1^	Production Size	Usage	Source
*sptP*-cat-F	GTTTGCTGATTAATTGGAATGCTGCTGACCGCAAATCGTGCAGGCCCAGttacgccccgccctgccac	960 bp	*sptP* gene homologous arms	This study
*sptP*-cat-R	AGAAAATAGAACCGGCGCGCCAATGCCACAGACGATGAGCGGACCGCAtacctgtgacggaagatcacttc			
*sptP*-out-F	GTACGAACCGCTAATGCCACAGG	1145 bp or 285 bp	Identification of *sptP* mutant	This study
*sptP*-out-R	GAGAGGTGGTTGTAAAGCTCTACTCATG			

^1^ Lowercase letter: Cmr cassette amplification.

**Table 2:** Median lethal dose of ATCC14028 and ATCC14028Δ*sptP* injected intraperitoneally in mice.

Groups	Challenge Dose (CFUs)	Number of Dead Mice/Total Number of Mice	Mortality	LD_50_ (CFUs)
ATCC14028	10^5^	0/5	0%	5.01 × 10^5^
	10^6^	4/5	80%	
	10^7^	5/5	100%	
	10^8^	5/5	100%	
	10^9^	5/5	100%	
	10^10^	5/5	100%	
ATCC14028Δ*sptP*	10^5^	0/5	0%	2 × 10^7^
	10^6^	0/5	0%	
	10^7^	2/5	40%	
	10^8^	4/5	80%	
	10^9^	5/5	100%	
	10^10^	5/5	100%	
PBS	-	0/10	0%	-

## Data Availability

The datasets used and/or analyzed during the current study are available from the corresponding author upon reasonable request.

## Supplementary Materials

The following supporting information can be downloaded at: https://www.mdpi.com/article/10.3390/vaccines13020150/s1, Figure S1. Biological characteristic test of ATCC14028Δ*sptP*. (**A**) Genetic stability of ATCC14028Δ*sptP*. (**B**) Colony morphology of ATCC14028Δ*sptP* (a) and ATCC14028 (b); Table S1. Biochemical properties of *Salmonella Typhimurium* ATCC14028 and ATCC14028Δ*sptP*.
