# Supplementary material for: Characterization and Protective Efficacy of a Salmonella Typhimurium ATCC 14028 sptP Mutant as a Live Attenuated Vaccine Candidate
Source: Vaccines (Basel). 2025 Jan 31;13(2):150. doi: 10.3390/vaccines13020150 (PMC11860608; doi:10.3390/vaccines13020150)
Supplement: Supplementary file 1 [file vaccines-13-00150-s001.zip › vaccines-3386770-supplementary.pdf]

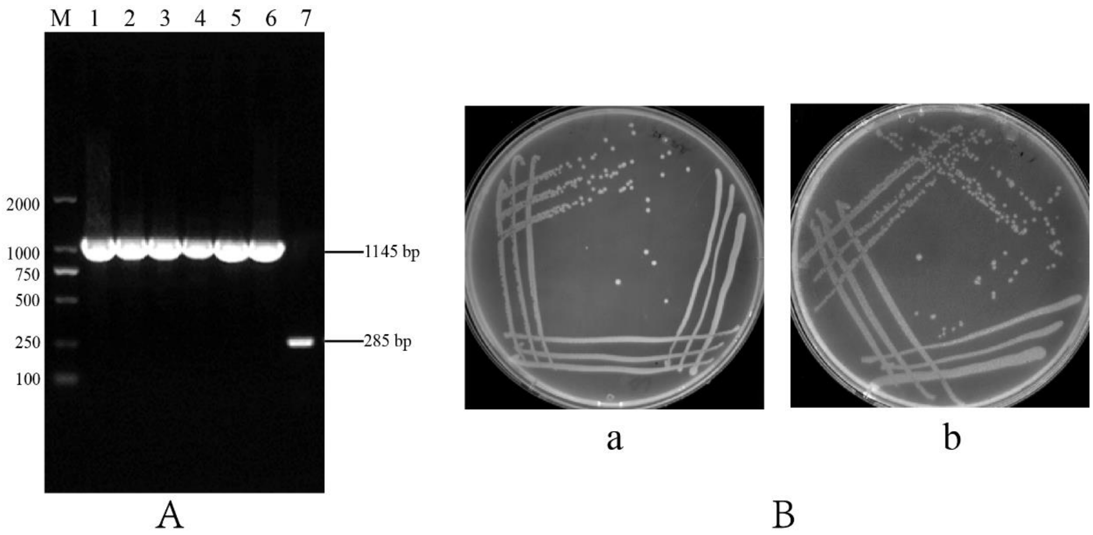

Supplementary Figure S1. Biological characteristic test of ATCC14028Δ*sptP*. (A) Genetic stability of ATCC14028Δ*sptP*. Lane M: DL2000 DNA Marker; Lanes 1-6: Genomic DNA of the 5th, 10th, 15th, 20th, 25th and 30th generation strains of ATCC14028Δ*sptP*; Lane 7: ATCC14028 genomic DNA. The PCR product length of ATCC14028Δ*sptP* is 1145 bp, and the PCR product length of ATCC14028 is 285 bp. (B) Colony morphology of ATCC14028Δ*sptP* (a) and ATCC14028 (b).

Table S1. Biochemical properties of *Salmonella* typhimurium ATCC14028 and ATCC14028Δ*sptP*

Biochemical properties	Strains	
	ATCC14028Δ*sptP*	ATCC14028
β-galactosidase enzyme	-	-
Lysine decarboxylase	+	+
Ornithine decarboxylase	+	+
H_2S	+	+
Urease	+	+
Indole	-	-
Fermentation of glucose	+	+
Fermentation of mannose	+	+
Fermentation of sorbitol	+	+
KCN（Potassium cyanide）	-	-
Citrate utilization	+	+

"+" represents positive, "-" represents negative.

Sequence 1: *sptP* gene

TCAGCTTGCCGTCGTCATAAGCAACTGGGCTTGCATTGCTTTTAGTTGTACAAACTGTGAGGCGTCTTCCAGCATT
CTATTGTTCCGTGAATCCCGGAAATCTGCACGTACCTGCTCCAGATTACTATGAGGATTATCCTTAAGTACAAGGG
CCGCCGCCATCGTTCCGGTTCTTCCCACTCCGCCCAGACAATGAATCATCGGTAAATGCTTATCTGATGAACTACG
CCCCGGCGCGCCATTTTGGTTACTATTTTTCACCCTATCCGCCAGGTATTCTAACTGATCCGTAGACGGTAACGGC
TGGTGATCTGGCCAATTTTTCACATGCAATACCGGGATTGTATACCGCTTTTCCCCGCAGGACAGTTGCATATTGT
ATTGGTCTATCGCTTCTCCCTGACTGGCTGAGCTCACTTTTTGGCTGTTGGTATGCACCTCGCCAAAGGTGTAGCT
CCCTCTGAAATAGGGTGGTAATTGTTTTGCCTGCATCTGATCTTCCGACGTTAACACCACCAGGCACGAGCATTCT
TTTTCAAGAAGCATTTTCATATGCGCTTCCAGCGCATCCGGCGTATTTTTTGGGTACGAACCGGCTAATGCCACAG
GCTTACCGTCAAAAGTTAACGTATTCACTGGCACAGGCATTCCATCGCTCAGTTTCACCTGGGTTTGCTGATTAAT
TGGAATGCTGCTGACCGCAAATCGTGCCAGGCCCAGTGTCGGTCCGCTCATCGTCTGTGGCATTGGCGCGCCGGCT
TCTATTTTCTCAAGTTCAGCTGTAACATTTTTCAGTTCTTTAGCAATAACGTGAATTTTTTTACAGCCTGGGTTA
ATTCATGAGTAGAGGCTTTATCAACCCACCTCTCAACCTCTCCTCCACAGGTTCCCCATTGTGAGAACCGGGCTAC
ACCGACCTGAATATTTGTTAACGTTGTAACATAATCATTAAGTTGTTTAGCCTCTGGAATTTTATTTAAATTCTGT
AAATTCGTCATTAATGAACGCAGCGGGCCGTTACCTGAAGCCATCTCCTGGAAGTTTTCTCGCAGGCTATTTCCAT
CCATTTGTTCCAGTTGCGGTAATGTTCTTTTAAGTCCCTTTAGCGCGATATCGAGTAAAGGTTGCTTACTTTCTGC
TCCAACATCGTTATTTTTTTCTGCCACTTTTGTATCGCCGCCTTTTATGACTAAAGCGGCATTCCTGACACCAACA
TTATCCTTGCTCTTAATAAGGTTTATAAACCCTTCGTCAGCAGCTTTTACACACTCCGTGATCTGCACTGCTAAAC
GTTGGGTAAGGGGTTTGTTCATATTTATACGGGACATTAACAGTGCGTCATTAACCGCTGTTTCCCCATATTTTTC
CGTTAGTGCATGGAGAAATGTCTGTAAAATCTTTTGGTCCTGTACTCTGATATTTTCCGTATGTTTTTGCACCACT
TCAGTGTTTTTAAATAACGGCATTTTTCCAAGCCAGGTTAATACTTTTGACGAAAATTTTTCAGGCGCAACATATG
CCTTATCAGTATTTTCCTTAGCAATATAAAGTCGGGCATCATTCGACACACCAACTTTTGAAAACGAAGACAACGT
TAAATTATTCAATTTTCTCTCCTCATACTTTAGCAT

Sequence 2: *sptP* gene targeting fragment

GTTTGCTGATTAATTGGAATGCTGCTGACCGCAAATCGTGCCAGGCCCAGttacgccccgccctgccactcatcgc
agtactgttgtaattcattaagcattctgccgacatggaagccatcacagacggcatgatgaacctgaatcgccag
cggcatcagcaccttgtcgccttgcgtataatatttgcccatggtgaaaacgggggcgaagaagttgtccatattg
gccacgtttaaatcaaaactggtgaaactcacccagggattggctgagacgaaaaacatattctcaataaaccctt
tagggaaataggccaggttttcaccgtaacacgccacatcttgcgaatatatgtgtagaaactgccggaaatcgtc
gtggtattcactccagagcgatgaaaacgtttcagtttgctcatggaaaacggtgtaacaagggtgaacactatcc
catatcaccagctcaccgtctttcattgccatacggaattccggatgagcattcatcaggcgggcaagaatgtgaa
taaaggccggataaaacttgtgcttatttttctttacggtctttaaaaaggccgtaatatccagctgaacggtctg
gttataggtacattgagcaactgactgaaatgcctcaaaatgttctttacgatgccattgggatatatcaacggtg
gtatatccagtgatttttttctccattttagcttccttagctcctgaaaatctcgataactcaaaaaatacgcccg
gtagtgatcttatttcattatggtgaaagttggaacctcttacgtgccgatcaacgtctcattttcgccaaaagtt
ggcccagggcttcccggtatcaacagggacaccaggatttatttattctgcgaagtgatcttccgtcacaggtaTG
TCGGTCCGCTCATCGTCTGTGGCATTGGCGCGCCGGCTTCTATTTTCT

Sequence 3: partial sequence of the region where the sptp gene is replaced by the cm resistance gene

TCAGCTTGCCGTCGTCATAAGCAACTGGGCTTGCATTGCTTTTAGTTGTACAAACTGTGAGGCGTCTTCCAGCATT
CTATTGTTCCGTGAATCCCGGAAATCTGCACGTACCTGCTCCAGATTACTATGAGGATTATCCTTAAGTACAAGGG
CCGCCGCCATCGTTCCGGTTCTTCCCACTCCGCCCAGACAATGAATCATCGGTAAATGCTTATCTGATGAACTACG
CCCCGGCGCGCCATTTTGGTTACTATTTTTCACCCTATCCGCCAGGTATTCTAACTGATCCGTAGACGGTAACGGC
TGGTGATCTGGCCAATTTTTCACATGCAATACCGGGATTGTATACCGCTTTTCCCCGCAGGACAGTTGCATATTGT
ATTGGTCTATCGCTTCTCCCTGACTGGCTGAGCTCACTTTTTGGCTGTTGGTATGCACCTCGCCAAAGGTGTAGCT
CCCTCTGAAATAGGGTGGTAATTGTTTTGCCTGCATCTGATCTTCCGACGTTAACACCACCAGGCACGAGCATTCT
TTTTCAAGAAGCATTTTCATATGCGCTTCCAGCGCATCCGGCGTATTTTTTGGGTACGAACCGGCTAATGCCACAG
GCTTACCGTCAAAAGTTAACGTATTCACTGGCACAGGCATTCCATCGCTCAGTTTCACCTGGGTTTGCTGATTAAT
TGGAATGCTGCTGACCGCAAATCGTGCCAGGCCCAGttacgccccgccctgccactcatcgcagtactgttgtaat
tcattaagcattctgccgacatggaagccatcacagacggcatgatgaacctgaatcgccagcggcatcagcacct
tgtcgccttgcgtataatatttgcccatggtgaaaacgggggcgaagaagttgtccatattggccacgtttaaatc
aaaactggtgaaactcacccagggattggctgagacgaaaaacatattctcaataaaccctttagggaaataggcc
aggttttcaccgtaacacgccacatcttgcgaatatatgtgtagaaactgccggaaatcgtcgtggtattcactcc
agagcgatgaaaacgtttcagtttgctcatggaaaacggtgtaacaagggtgaacactatcccatatcaccagctc
accgtctttcattgccatacggaattccggatgagcattcatcaggcgggcaagaatgtgaataaaggccggataa
aacttgtgcttatttttctttacggtctttaaaaaggccgtaatatccagctgaacggtctggttataggtacatt
gagcaactgactgaaatgcctcaaaatgttctttacgatgccattgggatatatcaacggtggtatatccagtgat
ttttttctccatttttagcttccttagctcctgaaaatctcgataactcaaaaaatacgcccggtagtgatcttatt
tcattatggtgaaagttggaacctcttacgtgccgatcaacgtctcattttcgccaaaagttggcccagggcttcc
cggtatcaacagggacaccaggatttatttattctgcgaagtgatcttccgtcacaggtaTGTCGGTCCGCTCATC
GTCTGTGGCATTGGCGCGCCGGCTTCTATTTTCTCAAGTTCAGCTGTAACATTTTTCAGTTCTTTAGCAATAACGT
GAATTTTTTTTACAGCCTGGGTTAATTCATGAGTAGAGGCTTTATCAACCCACCTCTCAACCTCTCCTCCACAGGT
TCCCCATTGTGAGAACCGGGCTACACCGACCTGAATATTTGTTAACGTTGTAACATAATCATTAAGTTGTTTAGCC
TCTGGAATTTTATTTAAATTCTGTAAATTCGTCATTAATGAACGCAGCGGGCCGTTACCTGAAGCCATCTCCTGGA
AGTTTTCTCGCAGGCTATTTCCATCCATTTGTTCCAGTTGCGGTAATGTTCTTTTAAGTCCCTTTAGCGCGATATC
GAGTAAAGGTTGCTTACTTTCTGCTCCAACATCGTTATTTTTTTCTGCCACTTTTGTATCGCCGCCTTTTATGACT
AAAGCGGCATTCCTGACACCAACATTATCCTTGCTCTTAATAAGGTTTATAAACCCTTCGTCAGCAGCTTTTACAC
ACTCCGTGATCTGCACTGCTAAACGTTGGGTAAGGGGTTTGTTCATATTTATACGGGACATTAACAGTGCGTCATT
AACCGCTGTTTCCCCATATTTTTCCGTTAGTGCATGGAGAAATGTCTGTAAAATCTTTTGGTCCTGTACTCTGATA
TTTTCCGTATGTTTTTGCACCACTTCAGTGTTTTTAAATAACGGCATTTTTCCAAGCCAGGTTAATACTTTTGACG
AAAATTTTTCAGGCGCAACATATGCCTTATCAGTATTTTCCTTAGCAATATAAAGTCGGGCATCATTCGACACACC
AACTTTTGAAAACGAAGACAACGTTAAATTATTCAATTTTCTCTCCTCATACTTTAGCAT

Red letters: *sptP* gene coding frame.

Double underlined: upstream homologous recombination arm.

Double underlined: downstream homologous recombination arm.

Wavy underline: knockout site outer primers.

Framed letters: gene overlapping sequence.

small letters: cm resistance gene sequence, derived from pACYC184 plasmid.
